# Natural *Bletilla striata* Polysaccharide-Based Hydrogels for Accelerating Hemostasis

**DOI:** 10.3390/gels11010048

**Published:** 2025-01-08

**Authors:** Hui-Fang Lin, Yue-Yue Wang, Feng-Zhen Liu, Zi-Wei Yang, Hao Cui, Si-Yu Hu, Feng-He Li, Pei Pan

**Affiliations:** School of Pharmacy, Anhui Medical University, Hefei 230032, China

**Keywords:** *Bletilla striata* polysaccharide, hydrogels, natural, borate ester bonds, hemostasis

## Abstract

Most of the existing hydrogel dressings have inadequacies in mechanical performance, biological activities, compatibility, or versatility, which results in the development of rapid, green, and cost-effective approaches for hydrogels in biochemical and biomedical applications becoming a top-priority task. Herein, inspired by the inherent bioactivity, water retention properties, and biocompatibility of natural polysaccharide hydrogels, we have prepared self-healing gels. Using *Bletilla striata* polysaccharide (BSP), carboxymethyl chitosan (CMCS), and borax via borate ester linkages, we created hemostatic and self-healing Chinese herbal medicine hydrogels in varying concentrations (2.5%, 3.0%, and 4.0%). A rotational rheometer was used to describe the hydrogels’ shape and rheological characteristics. At all concentrations, it was found that the hydrogels’ elastic modulus (G′) consistently and significantly outperformed their viscous modulus (G″), suggesting a robust internal structure. All of the hydrogels had cell viability levels as high as 100% and hemolysis rates below 1%, indicating the hydrogels’ outstanding biocompatibility. Furthermore, the hydrogels demonstrated superior hemostatic qualities in an in vivo mouse tail amputation model, as well as in in vitro coagulation tests. The results show that the hydrogel possesses excellent self-healing properties, as well as a good biocompatibility and hemostatic performance, thus paving the way for the development of a potential hemostatic green hydrogel.

## 1. Introduction

Made of natural or synthetic polymers, hydrogels have a special three-dimensional network structure that exhibits high water absorption and biodegradability [1]. Hydrogels are one of the most promising types of biomaterials because of their high water content, which results in outstanding biocompatibility comparable to that of hydrated biological tissues [2]. Hydrogels, in particular, can provide callus and epithelial cells with a wet environment, which can hasten the establishment of collagen and fibroblast proliferation [3]. However, most hydrogel dressings rely on synthetic polymers, which involve complex and potentially harmful production methods [4]. In contrast, hydrogels containing an active ingredient derived from natural Chinese herbal medicine possess superior biocompatibility and unique biological properties compared to synthetic polymers [5]. In our research, we have advanced the development of a biocompatible hydrogel wound dressing. This was achieved by exclusively incorporating natural components and employing an eco-friendly chemical synthesis approach [6].

One of the main active ingredients of *Bletilla striata* (BS) is *Bletilla striata* polysaccharide (BSP), a gel-like polymer primarily made of glucomannan [7,8]. BSP is abundant and easy to obtain from medicinal resources, thus emerging as a promising source for biomaterials [9,10,11,12]. In recent studies, it has been revealed that BSP exhibits a range of therapeutic properties, including wound healing, anti-inflammatory, hemostatic, antibacterial, anti-fibrosis, antiviral, and promoting cell growth [13,14,15,16,17]. Moreover, this polysaccharide has long-lasting moisturizing effects. Its numerous hydroxyl groups readily engage in hydrogen bonding, endowing it with exceptional water affinity and contributing to high elasticity in BSP-based materials [18]. Despite the high biocompatibility and hemostatic effects of the BSP hydrogel, it is hampered by weak rheological properties. Consequently, CMCS was introduced to enhance its rheological performance. CMCS, as a prevalent derivative of chitosan, possesses excellent water solubility, biocompatibility, and antibacterial activity [19,20]. Therefore, it is widely utilized in wound dressings. The study revealed that the integration of CMCS within the poly vinyl alcohol (PVA) matrix significantly improves its biological compatibility, bolsters its mechanical attributes, and enhances its capacity for water absorption under ambient conditions [21]. With the low toxicity, low cost, and good water solubility of borax, the borate ester bond’s structure has been widely used in the development of self-healing hydrogels [22,23,24]. The plentiful hydroxyl groups present in BSP are instrumental in catalyzing the formation of borate linkages, which is essential for the successful creation of hydrogels [25,26]. The formation of borate ester bonds presents an opportunity to synthesize chemically crosslinked hydrogels without the need for catalysts, initiators, or coupling agents, thereby maintaining the biocompatibility of the resultant material [27].

**Figure 1:**
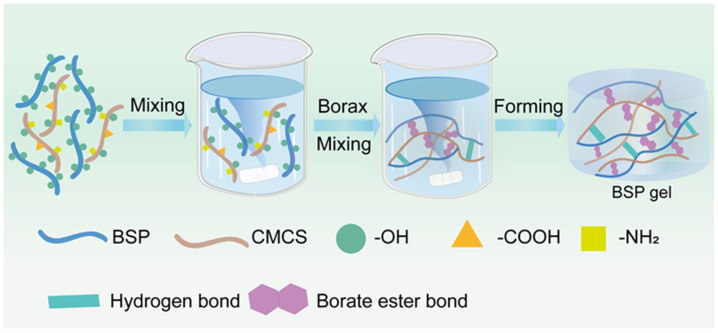
Schematic diagram of the preparation of BSP hydrogel.

Based on the current research status, we have successfully constructed a natural Chinese medicine hydrogel that is endowed with bioactivity and, at the same time, possesses self-healing hemostatic efficacy (Figure 1). On one hand, the extensive quantity of hydroxyl groups present on BSP engage in cross-linking reactions with boron atoms in borax, thereby leading to the formation of borate ester bonds. On the other hand, CMCS, which is equipped with amino and carboxyl groups, is capable of forming hydrogen bonds with borax [28,29]. This interaction serves to fortify and stabilize the hydrogel network. Owing to the inherent pharmacological properties of BSP, it is enabled to fulfill crucial functions such as hemostasis [30]. In this manner, a seamless integration is achieved between traditional Chinese medicine and hydrogel-based wound dressings, opening up new avenues for advanced medical applications.

## 2. Results and Discussion

### 2.1. Synthesis and Characterization of BSP Hydrogel

**Figure 2:**
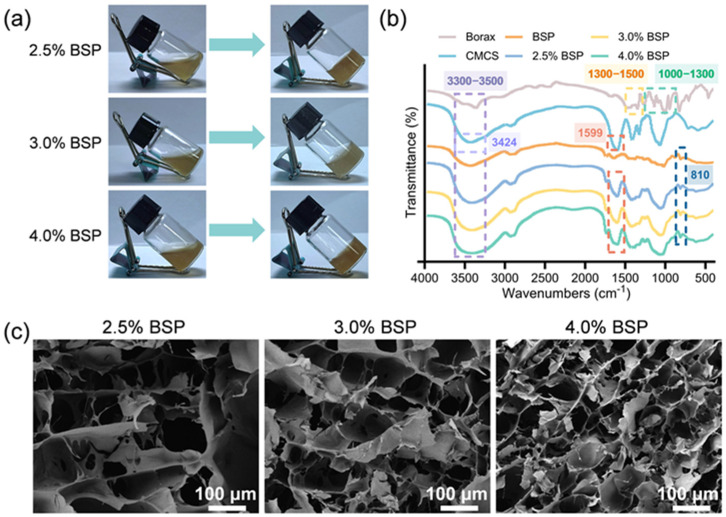
(**a**) Optical images of the hydrogel gelation; (**b**) FTIR spectra of BSP, CMCS, borax, and BSP hydrogel; (**c**) Representative SEM images of BSP hydrogels (scale bar: 100 μm).

Under physiological conditions, the hydrogel network was established via the formation of dynamic borate ester bonds. To prepare 2.5%, 3.0%, and 4.0% BSP solutions, 25 mg, 30 mg, and 40 mg of BSP were weighed out, respectively, and dissolved in 1 mL of deionized water under vigorous stirring (at 500 rpm) to form a transparent solution. Subsequently, 8 mg of CMCS was added and mixed until it was completely dissolved. After adding 10 mg of borax, a large number of hydroxyl groups from BSP combined with the boron ions of borax to form borate esters [18]. In addition, due to the formation of hydrogen bonds between the hydroxyl groups on BSP, as well as the amino, carboxyl, and hydroxyl groups on CMCS [31], the solution transformed from a liquid state to a gel state. (Figure 2a). These videos showed the state of the hydrogel after gelation (Videos S1 and S2).

As depicted in Figure 2b, Fourier transform infrared spectroscopy (FTIR) analysis disclosed specific peaks associated with BSP. The absorption peak at 3422 cm^−1^ was attributed to the hydroxyl groups present in BSP. The peak observed at 2925 cm^−1^ corresponded to the stretching vibrations of the methyl or methylene carbon groups within BSP. The region between 1000 and 1200 cm^−1^ captured the stretching vibrations and C-O-C glycosidic bonds of the C-O-H end groups. The characteristic absorption peak at 810 cm^−1^ signified the presence of mannose. In the case of borax, the absorption band ranging from 1000 to 1300 cm^−1^ was due to the in-plane bending vibrations of O-H, while the band from 1300 to 1500 cm^−1^ was ascribed to the asymmetric stretching vibrations of the BO_3_ group. For CMCS, the absorption peak at 3424 cm^−1^ corresponded to the combined stretching vibrations of the O-H and N-H groups. Meanwhile, the asymmetric stretching vibration of -COO^−^ was linked to the absorption peak at 1599 cm^−1^. The BSP hydrogels exhibited novel absorption peaks at 1417 cm^−1^, which were ascribed to the asymmetric stretching vibrations characteristic of the B-O-C bonds within the BSP–boronate complex. The infrared spectroscopic analysis of BSP hydrogels revealed characteristic peaks associated with BSP, CMCS, and borax, indicating the successful integration of these components. The spectra of the three hydrogel formulations were largely similar, which supports the formation of BSP hydrogels. Additionally, the microstructure of the freeze-dried BSP hydrogel was further characterized using scanning electron microscopy (SEM), with cross-sectional views depicted in Figure 2c. The lyophilized BSP hydrogel exhibits a uniformly porous structure, which is beneficial for efficient drug loading and cell recruitment. As the content of BSP increased, the average pore sizes of the hydrogels decreased successively, and they were 50.2 ± 12.14 μm, 38.1 ± 9.61 μm, and 19.06 ± 3.05 μm (Figure S1), respectively. In addition, the 4.0% BSP hydrogel has the smallest porosity, at approximately 87.22% (Figure S2). This might be due to the fact that more borate ester bonds formed between the hydroxy groups of BSP and borax, which increased the degree of crosslinking of the hydrogel. The results obtained suggest that we have successfully fabricated BSP hydrogels incorporating borate ester bonds.

### 2.2. Rheological Behaviors of BSP Hydrogels

**Figure 3:**
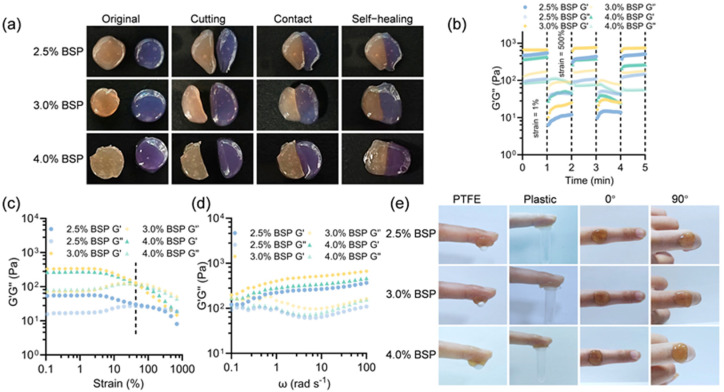
(**a**) Macroscopic assessment of the hydrogel’s capacity for self-healing. (**b**) The step-strain behavior of hydrogels with high (500%) to low (1%) strains. (**c**) The strain sweep measurement of hydrogels at 0.1~1000% strain. (**d**) The dynamic frequency sweep measurements of hydrogels at 1% strain. (**e**) Adhesive capacity on different substrates.

Due to the reversible nature of the borate ester bonds within the structure, the BSP hydrogel that was synthesized demonstrates superior injectability and self-healing ability. Chronic wounds often present irregular skin defects. Wound dressings with injectability can effectively fill irregular wound beds and improve treatment outcomes [32]. As illustrated in Figure S3, a syringe was used to continuously inject the BSP hydrogel to form the letters “AHMU”, indicating the injectability of the hydrogel. The injectability of the BSP hydrogel deteriorates as the concentration increases. A concentration of 2.5% yields the best injectability. There is a resistance when injecting hydrogels at 3.0% and 4.0% BSP concentrations, especially at 4.0% BSP. In practical applications, hydrogels with rapid self-healing capabilities can effectively accommodate the recurrent cracking of wounds caused by intense physical activity, eliminating the need for cumbersome replacement procedures and exhibiting an extended lifespan [9,33]. To assess the BSP hydrogel’s capacity for self-healing, a macroscopic healing test was conducted (Figure 3a). The circular hydrogels stained with crystal violet and those of the original color were severed into halves, respectively. They were then gradually reattached to the other stained semicircular gels. After a re-integration period of 4 min, the reassembled hydrogels with concentrations of 2.5% and 3.0% were healed into an integrated piece in the original shape, without any disruptions or crack lines. However, the 4.0% BSP hydrogel could achieve self-healing only after 8 min. Subsequently, continuous cyclic strain tests on the BSP hydrogel were carried out using a rheometer by applying two cycles of 1% and 500% strain. The 3.0% and 4.0% BSP hydrogels showed superior self-healing properties. As illustrated in Figure 3b, when a 500% strain was applied, the storage modulus (G′) value of the hydrogels dropped below the loss modulus (G″), indicating the collapse of the network within the hydrogels. Nevertheless, when the strain was restored to 1%, G′ nearly returned to its initial level, suggesting the restoration of the network structure. Ulteriorly, this result confirmed the excellent self-repairing ability of the hydrogel. Additionally, other rheological characteristics of the hydrogels were also detected. Strain scanning was used to assess the critical values at which the collapse of the network of the hydrogels occurs. Both of them showed G″ greater than G′ at a strain ≈60%, indicating the rupture of an elastic network of the hydrogels and its change into a liquid-like state (Figure 3c). Figure 3d demonstrated that all the experimental hydrogel groups demonstrated the formation of an internal elastic network within the hydrogels within a certain frequency range, where G′ was higher than G″. The borate ester bond imparts adhesive surface properties to the hydrogel. This is attributed to the fact that the abundant phenolic hydroxyl groups have the ability to bind with organ tissues. The adhesion characteristics of the hydrogel with respect to materials and tissues were explored. As shown in Figure 3e, the hydrogels showed firm adhesion to skin, polytetrafluoroethylene (PTFE), and plastic. In addition, the hydrogel demonstrated the ability to bond with fingers, remaining in place without detaching during flexion from 0 to 90 degrees. The combination of the self-healing, injectable, and adhesive characteristics of the hydrogel might be beneficial in protecting wounds from drug injury during exercise and dynamic wound care. Furthermore, we attached the hydrogel to the wrist and subjected it to movements at various angles (Figure S4). After multiple repetitions of motion (Video S3), the hydrogel still adhered to the wrist, further demonstrating its excellent adhesion properties. Additionally, we further utilized a rheometer to measure the relationship between the lateral force and gap required by the hydrogel (Video S4), reflecting its adhesion performance. The 4.0% BSP hydrogel demonstrated the best adhesion properties (Figure S5).

It is widely recognized that hydrogel-based dressings possess the ability to retain moisture on wound surfaces, thereby creating a hydrated environment conducive to wound healing through angiogenesis and cell proliferation. As a result, this facilitates optimal wound healing. To evaluate the moisture retention properties of various dressings, an examination of the water evaporation rate from several hydrogels was conducted. The experimental results, as depicted in Figure S6, show a time-dependent decline in moisture content among three distinct hydrogel formulations within 22 h, resulting in an approximately 40% reduction in water content. Notably, the 4.0% BSP hydrogel demonstrated a slightly lower water retention capacity compared to other formulations. Specifically, the hydrogels exhibited a consistent water loss of about 3% every two hours, highlighting their strong moisture retention properties. According to previous research, BSP hydrogel has a better water retention performance than some BSP-based hydrogels, including the BSP/BER hydrogel made by Chen et al. [34] and the oxidized BSP-based aerogel (ORBPS/PVA) made by Yan et al. [18]. These findings are crucial for the development of advanced wound dressings that can provide prolonged hydration to the wound bed and potentially enhance the healing process. The comparative analysis of different hydrogel formulations offers valuable insights for the selection of materials with optimal moisture retention characteristics for therapeutic applications. Figure S7 shows that the hydrogel has a good swelling ratio and can absorb the exudate from the wound site. Effective degradation is crucial to prevent the retention of residual hydrogel in deep wounds, which could impede the healing due to its tissue adaptability. The in vitro degradation analysis demonstrated that the BSP hydrogel could degrade by 80% within 24 h. Additionally, the degradation rates of hydrogels at different concentrations do not differ much. Initially, the 4.0% BSP hydrogel has the slowest degradation rate (Figure S8).

### 2.3. In Vitro Biocompatibility of BSP Hydrogels

**Figure 4:**
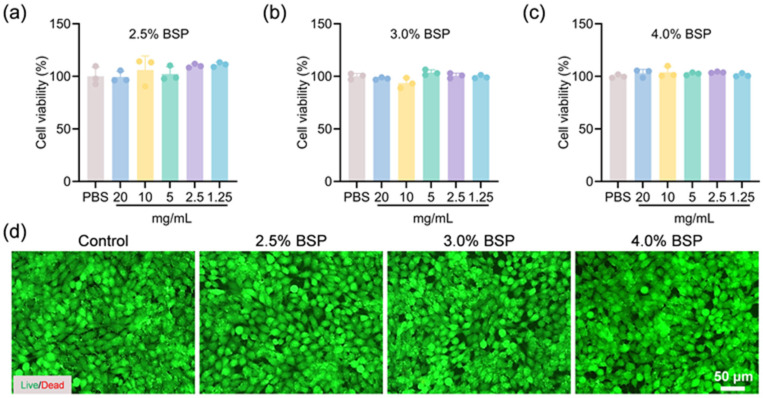
(**a**–**c**) The viability of L929 cells cultured with BSP hydrogel extracts at 2.5%, 3.0%, and 4.0% concentrations (*n* = 3). (**d**) The corresponding live/dead staining images of these cells. Live cells are indicated by green fluorescence from Calcein-AM (AM), and dead cells are marked by red fluorescence from propidium iodide (PI).

Biocompatibility is an essential characteristic of biomaterials which come into indirect contact with tissues and blood. Compatibility with cells and blood is acknowledged to be an essential criterion for the application of hydrogels in wound care [35]. To assess the cytocompatibility of the BSP hydrogel, we utilized both the 3-(4,5-Dimethylthiazol-2-yl)-2,5-diphenyltetrazolium bromide (MTT) assay and live/dead staining protocols, employing L929 fibroblasts as the cellular model for this evaluation. The biocompatibility of BSP hydrogels was evaluated using the extraction method to test for cytotoxicity. Data presented in Figure 4a–c demonstrate that the viability of cells was consistent across various hydrogel concentrations, comparable to that of the control group in media supplemented with hydrogel extracts. This consistency suggests that the hydrogels possess a high degree of compatibility with cells. As shown in Figure 4d, fluorescent imaging revealed that most cells exhibited continuous proliferation, maintaining their typical spindle-shaped morphology. Upon comparison with the control group, a minimal number of red-labeled dead cells was observed, with no significant differences detected, suggesting that the cells are in a normal state of proliferation. The experimental outcomes indicated that the hydrogel exhibits superior compatibility with cellular environments.

### 2.4. In Vitro Whole Blood Clotting Test and In Vivo Hemostasis of Hydrogels

**Figure 5:**
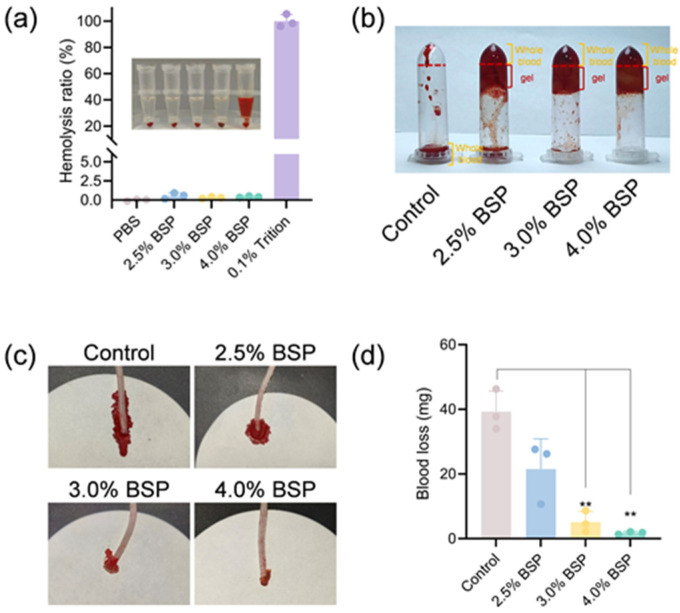
(**a**) The hemolysis ratio of hydrogels (*n* = 3). (**b**) Hemostasis process images of hydrogels. (**c**) Bloodstain photographs of the mouse tail amputation model (*n* = 5). (**d**) Quantitative analysis regarding blood loss in the mouse tail model (*n* = 3, ** *p* < 0.01).

The blood compatibility of BSP hydrogels was determined using an in vitro hemolysis test. Following incubation under in vitro conditions that mimic physiological settings, the visual and color characteristics of three distinct hydrogel groups and a positive control group containing 0.1% Triton X-100 were examined, as depicted in Figure 5a. All hydrogel samples exhibited a color identical to that of the PBS, whereas the positive control group displayed a vivid red hue. Quantitative analysis revealed that the hemolytic rates for all hydrogel formulations were below the 5% threshold. Specifically, the hemolytic rates for the 2.5%, 3.0%, and 4.0% BSP hydrogels were recorded at 0.56%, 0.29%, and 0.41%, respectively. These findings suggest that the hydrogels exhibit favorable hemocompatibility. In the early stage of wound healing, hemostasis plays a pivotal role as a fundamental process by forming a provisional barrier to safeguard underlying tissues from further damage and the threat of infection. This initial response is of critical importance for initiating the subsequent stages of the healing cascade. In view of the common occurrence of bleeding in both surgical and traumatic wounds, advanced wound dressings are required to be capable of accelerating the hemostasis process. We evaluated the hemostatic performance of hydrogels by simulating an in vitro clotting (Figure 5b). It is observed that within the first 60 s, blood in the BSP hydrogel coagulates at the bottom of the test tube. When inverted, there is no blood flowing out, indicating rapid hemostasis. Particularly, the 4.0% BSP hydrogel shows this characteristic. The coagulatory potential of the BSP hydrogel was rigorously examined through a mouse tail amputation model; the results are graphically represented in Figure 5c. This result demonstrated that the 4.0% BSP hydrogel significantly mitigated hemorrhage when juxtaposed with the control group, as further elaborated in Figure 5d. The hydrogel’s in vivo hemostatic influence is predominantly attributed to the inherent coagulative attributes of BSP and its rapid formation of a physical barrier at the site of injury, effectively staunching blood flow. This study underscores the BSP hydrogel’s promise as an effective hemostatic agent.

## 3. Conclusions

In conclusion, taking advantage of the benefits offered by traditional Chinese medicine, a set of BSP hydrogels with exceptional self-healing capabilities has been successfully synthesized. This accomplishment is made possible through the establishment of borate ester linkages between BSP and borax. The components of Chinese herbal medicine and materials not only avoid the adverse effects of chemical processing but also maintain the efficacy of traditional Chinese medicine, thereby ensuring the biocompatibility of the hydrogel. These hydrogels have a stable porous network structure and show outstanding self-healing and water retention properties. Among them, the 2.5% BSP hydrogel and 3.0% BSP hydrogel have relatively good water retention properties. Moreover, the 2.5% hydrogel has good self-healing abilities. In addition, the 4.0% BSP hydrogel has the best hemostatic performance. Different concentrations of BSP hydrogel can be selected according to different applications. Significantly, BSP hydrogels demonstrate extraordinary potential in hemostasis, providing a novel direction for the development of Chinese herbal hydrogels. This research paves the way for the application of hydrogels in the field of medicine, offering new ideas for the integration of traditional Chinese medicine and modern materials science. However, extensive in vivo studies are still required to confirm the efficacy and safety of the hydrogels in wound models, as well as their impact on inflammatory and healing properties, which will be the focus of our future research.

## 4. Materials and Methods

Materials: *Bletilla striata* polysaccharide (BSP) and borax were purchased from Yuanye Bio-Technology (Shanghai, China). Carboxymethyl chitosan (CMCS) was purchased from Macklin Biochemical Co., Ltd. (Shanghai, China). 3-(4,5-Dimethyl-2-thiazolyl)-2,5-diphenyl tetrazolium bromide (MTT) was obtained from Energy Chemical Technology (Shanghai, China). Mouse fibroblasts (L929 cells) were obtained from the Cloud-Clone Corp. (Wuhan, China).

Animals: Twenty male BALB/c mice weighing approximately 20 g were used for the animal experiments. The mice were housed in a specific pathogen-free environment and maintained on a 12 h light/dark cycle.

Preparation of BSP hydrogels: A total of 25 mg, 30 mg, and 40 mg of BSP was weighed, respectively, and dissolved in ultrapure water to prepare 1 mL of BSP solutions with mass fractions of 2.5, 3.0, and 4.0%. Subsequently, 8 mg of CMCS was added to form a hydrogel precursor solution. Three precursor hydrogel solutions were mixed at room temperature until homogeneous and transparent composite hydrogels were created, after which, 10 mg of borax was added to each solution.

Fourier transform infrared spectroscopy (FTIR): The Fourier transform infrared (FT-IR) spectra of borax, BSP, CMCS, and BSP-based hydrogels were obtained utilizing a Fourier transform infrared spectrometer (Thermo, Nicolet 6700, USA). The spectral data were acquired across a wavenumber range spanning from 4000 to 400 cm^−1^.

Scanning electron microscopy (SEM): A German-made ZEISS Sigma360 scanning electron microscope (SEM) was employed to inspect the microstructures of the lyophilized hydrogel specimens that were fabricated at concentrations of 2.5%, 3.0%, and 4.0% BSP.

Self-healing ability of the hydrogel: To assess the hydrogel’s capacity for self-healing, a macroscopic examination was performed. Hydrogel samples, distinguished by their distinct colors, were sectioned into two separate parts. These parts were then carefully aligned and pressed together to achieve full contact across the cut surface. It was observed that the hydrogel exhibited a noticeable self-healing response within a short duration of 10 min. Photographic documentation was undertaken throughout this process to capture the progression of self-healing.

The injectability of the hydrogel: The hydrogels were loaded into a syringe and extruded. The injectability of the hydrogels was assessed visually.

Water loss rate: Before being moved into a centrifuge tube for additional testing, the hydrogel’s initial moisture content was measured and designated as M_1_. After that, the hydrogel-containing tube was placed inside an incubation chamber that was kept at a steady 37 °C. M_2_, the mass of the tube, was measured and recorded at regular intervals of two hours. The determination of the hydrogel’s water loss rate was computed in accordance with the following equation:

Water loss rate (%) = (M_1_ − M_2_)/M_1_ × 100%

Swelling ability: Tests of the hydrogel’s capacity to swell were carried out in phosphate-buffered saline (PBS) at 37 °C and a pH of 7.4 [12]. We measured the wet hydrogel’s initial weight and noted it as W_0_. At different points in time, the swelled hydrogels were extracted. Following the removal of surplus water using filter paper, the hydrogel’s weight was noted as W_t_. The formula for the swelling ratio is as follows: (W_t_/W_0_) × 100%.

Degradation rate: BSP hydrogels were submerged in 5 mL of phosphate-buffered saline (PBS, pH 7.4) containing 1000 U/mL of lysozyme for the purpose of in vitro degradation studies [26]. An incubator with a continuous temperature setting of 37 °C was used to incubate the hydrogels. The hydrogels were removed, freeze-dried, and weighed at intervals of 0, 6, 12, 18, and 24 h. The lysozyme solution was changed every day to keep the enzyme active. The following formula was used to determine the in vitro degradation rate:

Degradation rate = [(W_0_ − W_t_)/W_0_] × 100%

where W_0_ and W_t_ represent the weights of the original and remaining hydrogels, respectively.

Rheological properties and adhesion of the hydrogel: Referring to this method [12], we aimed to ascertain the mechanical properties of the BSP hydrogels. The storage modulus (G′) and loss modulus (G″) were quantified utilizing the Anton Paar MCR-302e rheometer. The assessment involved a frequency sweep test, conducted over a spectrum of 0.1 to 100 rad/s, while maintaining a strain level of 1%. Subsequently, a strain sweep test was executed at a constant frequency of 1 Hz, with the strain amplitude ranging from 0.01% to 100%, to determine the hydrogels’ response to varying degrees of deformation. To evaluate the self-healing ability of the hydrogel, a continuous step-strain experiment involving a minor strain (1%, 60 s) and a considerable strain (500%, 60 s) for two cycles was conducted.

The adhesion properties of hydrogels: The hydrogels were bonded to diverse substrate materials for investigating their adhesion performance to different substances. Subsequently, the hydrogels were directly affixed to finger joint regions. By bending the fingers to simulate movements in daily activities, the adhesion ability of the hydrogels to the skin was studied.

The cytocompatibility of the hydrogel: The impact of the hydrogel on cellular proliferation was assessed using MTT colorimetric assays and living/dead cell staining techniques. L929 cells were inoculated into a 96-well plate at a density of 5000 cells per well. Subsequently, the plate was incubated at 37 °C overnight to encourage cell adhesion. After 24 h, the initial growth medium was replaced with hydrogel extracts. The cells were then further incubated with these extracts for an additional 24 h period. Following an additional 24 h incubation period, MTT reagent was introduced to each well and allowed to react for 4 h. Subsequently, the optical density (OD) of the resulting formazan product was quantified at a wavelength of 570 nm using a spectrophotometric microplate reader.

A live/dead cell staining assay was performed utilizing L929 cells at a density of 20,000 cells per well. Following a 24 h period for cell adhesion to the plate, the cells were treated with hydrogel extracts for an additional 24 h. Subsequently, the cells were incubated with a Calcein-AM/PI staining solution for 25 min to distinguish between live and dead cells. The stained cells were then examined under a fluorescence microscope to evaluate the hydrogel’s effect on cell survival.

Hemolytic activity test of the hydrogel: Mouse blood was centrifuged for 10 min at 1000 rpm to separate the erythrocytes. Following three PBS buffer washes, the resulting erythrocytes were diluted with PBS to reach a final concentration of 5% (*v*/*v*). The centrifuge tube was filled with 500 µL of erythrocyte stock and 20 milligrams of hydrogel. It was then shaken for an hour at a speed of 200 rpm in an incubator set at 37 °C. The negative control was PBS buffer, whereas the positive control was 0.1% Triton X-100. The centrifuge tube’s contents were then centrifuged for ten minutes at 1000 rpm. A fresh 96-well microplate was then filled with 100 µL of the supernatant. A Molecular Devices microplate reader was used to record the solution’s absorbance at 540 nm. The following formula was used to obtain the hemolysis percentage:

Hemolysis (%) = [(A_p_ − A_b_)/(A_t_ − A_b_)] × 100%

where A_p_ represents the absorbance of the copolymer solution at a verified concentration, A_t_ is the absorbance of the positive control consisting of 0.1% Triton X-100, and A_b_ denotes the absorbance of the negative control, which was the PBS buffer.

In vitro hemostasis assay: Utilizing the method outlined in [26], we combined heparinized mouse blood and hemocytes with the control sample, as well as BSP hydrogels at concentrations of 2.5%, 3.0%, and 4.0%, respectively, in a volume ratio of 1:2. After a period of 1 min had passed, the centrifuge tube was inverted, and the hemostatic property was subsequently observed for analysis.

The mouse-tail amputation model: Based on the method outlined in [1], after anesthesia was administered, the mouse was securely placed on a surgical board. Surgical scissors were employed to cut off 50% of the tail length. To make sure that normal blood loss occurs, the mouse’s tail was left out in the open for 15 s following the cutting procedure. Subsequently, 100 milligrams of hydrogel was promptly applied to the bleeding site. At the conclusion of a five-minute post-application period, the soaked filter paper’s weight, now laden with absorbed blood, was precisely measured. This weight was then juxtaposed against the weight of filter paper from a control set, which had been kept separate from the hydrogel’s influence.

Statistics and reproducibility: Data analysis was performed utilizing GraphPad Prism software (10.1.2, GraphPad Software Inc., La Jolla, CA, USA). The thresholds for statistical significance were set at the following levels: * *p* < 0.05; ** *p* < 0.01; *** *p* < 0.001; ns—not significant.

## Data Availability

The original contributions presented in the study are included in the article/Supplementary Material; further inquiries can be directed to the corresponding author.

## Supplementary Materials

The following supporting information can be downloaded at: https://www.mdpi.com/article/10.3390/gels11010048/s1. Figure S1: Pore size distribution of BSP hydrogels with different concentrations; Figure S2: The porosity of BSP hydrogels with different concentrations; Figure S3: Visualization of the injectability of BSP hydrogels; Figure S4: Photographs of BSP hydrogels that were applied on the human wrist; Figure S5: The normal force-gap curves of hydrogels with different concentrations; Figure S6: The water loss rates of BSP hydrogels within 22 h at 37 °C; Figure S7: Swelling properties of BSP hydrogels; Figure S8: Degradation properties of BSP hydrogels; Video S1: The process of gel formation of the gel under stirring; Video S2: The post-gelation states of BSP hydrogels with varying concentrations; Video S3: The situation of BSP hydrogels applied on the human wrist after repeated movements; Video S4: The process of measuring the adhesiveness of hydrogels using a rotational rheometer.
