# Supplementary material for: Natural Bletilla striata Polysaccharide-Based Hydrogels for Accelerating Hemostasis
Source: Gels. 2025 Jan 8;11(1):48. doi: 10.3390/gels11010048 (PMC11764679; doi:10.3390/gels11010048)
Supplement: Supplementary file 1 [file gels-11-00048-s001.zip › gels-3354822-supplementary.pdf]

Supplementary Materials of:

Natural *Bletilla Striata* Polysaccharide-Based Hydrogels for Accelerating Hemostasis

Hui-Fang Lin[1], Yue-Yue Wang[1], Feng-Zhen Liu[1], Zi-Wei Yang[1], Hao Cui[1], Feng-He Li[1,*], Pei Pan[1,2,*]

[1]School of Pharmacy, Anhui Medical University, Hefei 230032, China

[2]Department of Gastroenterology, the Second Affiliated Hospital of Anhui Medical University, Hefei, Anhui Province, China

* Corresponding author

E-mail: peipan@whu.edu.cn

E-mail: lifenghe@ahmu.edu.cn

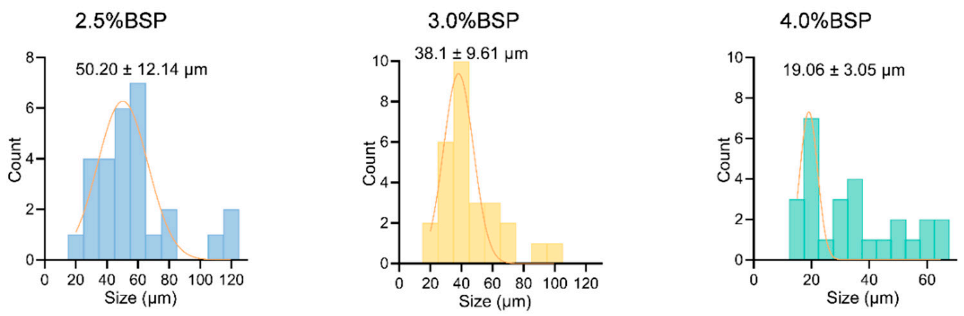

Figure S1: Pore sizes distribution of BSP hydrogels with different concentrations.

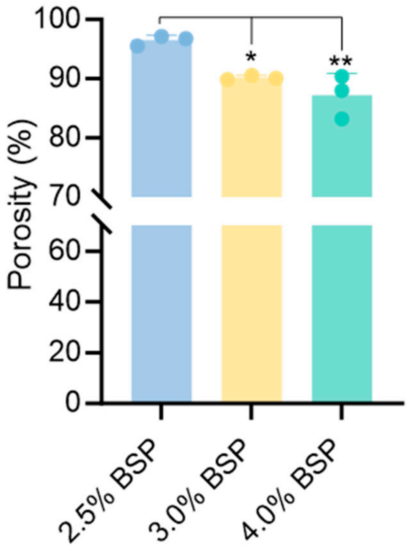

Figure S2: The porosity of BSP hydrogels with different concentrations.

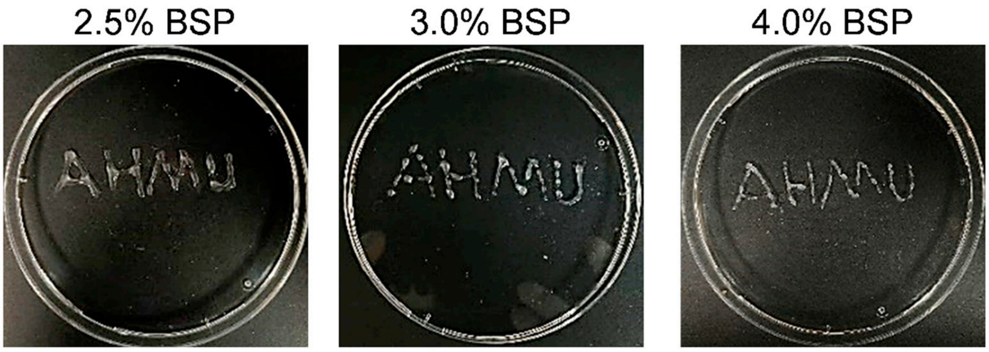

Figure S3: Visualization of the injectability of BSP hydrogels.

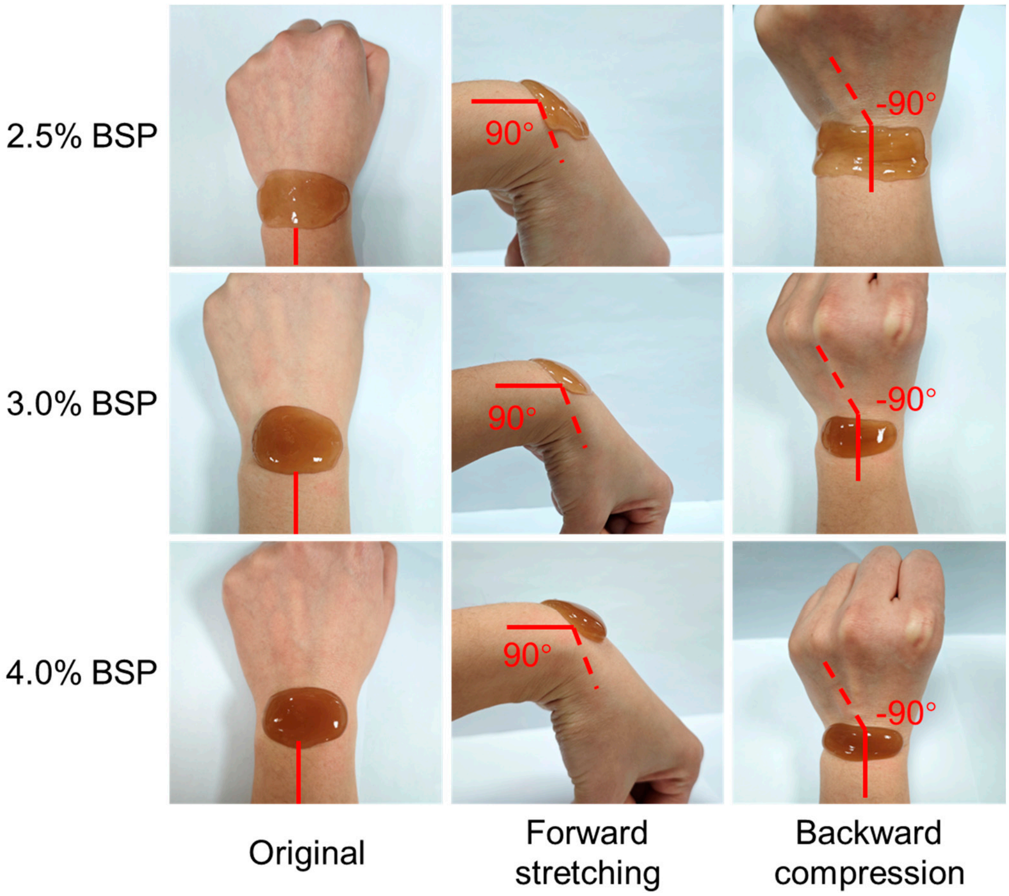

Figure S4: Photographs of BSP hydrogels that were applied on the human wrist.

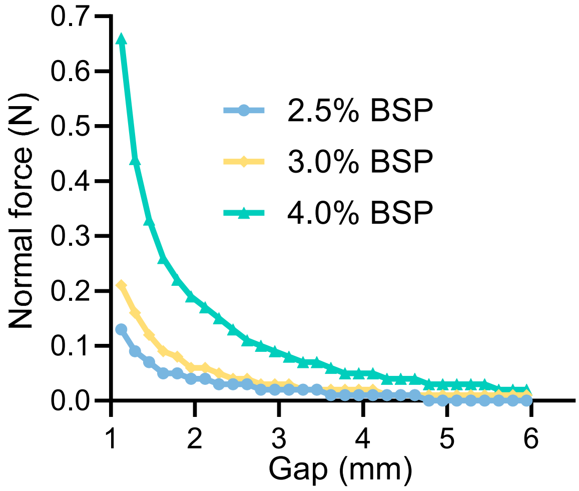

Figure S5: The normal force-gap curves of hydrogels with different concentrations.

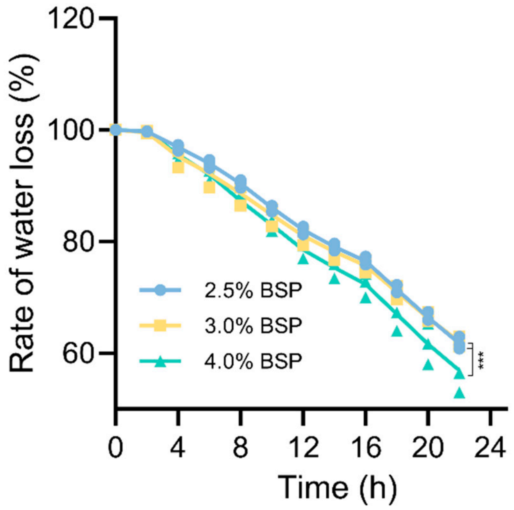

Figure S6: The water loss rates of BSP hydrogels within 22 h at 37 °C.

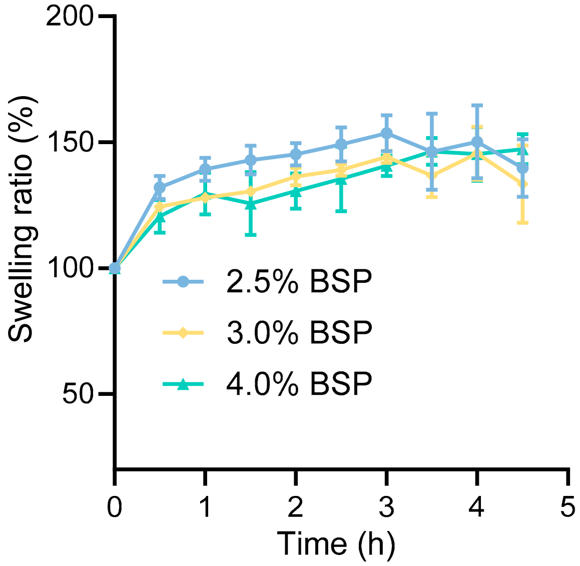

Figure S7: Swelling properties of BSP hydrogels.

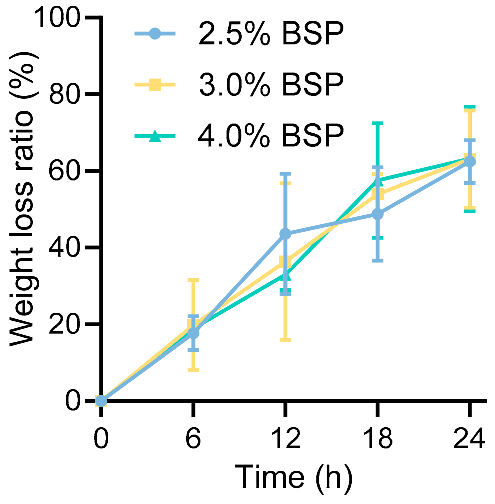

Figure S8: Degradation properties of BSP hydrogels.
